# The impact of pain on depression among middle-aged and older adult individuals in China: the chain mediation effect of self-rated health and life satisfaction

**DOI:** 10.3389/fpubh.2025.1546478

**Published:** 2025-04-24

**Authors:** Juju Huang, Yifan Gui, Keke Wang

**Affiliations:** Department of Psychosomatic Medicine, Jieshou People's Hospital, Fuyang, Anhui, China

**Keywords:** pain, depression, health status, life satisfaction, mediation

## Abstract

**Background:**

This research seeks to explore the association between pain and depression in middle-aged and older adult populations, with a particular focus on the mediating roles of self-rated health and life satisfaction within this framework.

**Methods:**

The study employed data from the 2020 China Health and Retirement Longitudinal Study. Utilizing linear regression modeling, we examined the primary determinants influencing depressive symptoms in the target demographic. Throughout the investigation, we applied Pearson correlation analysis to clarify the relationships among pain, depression, self-rated health, and life satisfaction. Additionally, the PROCESS 3.4 macro was utilized to evaluate the potential mediating effects of self-rated health and life satisfaction on the connection between pain and depression.

**Result:**

A significant negative correlation was observed between pain and both self-rated health and life satisfaction (*r* = −0.381, *p* < 0.01; *r* = −0.158, *p* < 0.01), while a notable positive correlation with depression was identified (*r* = 0.356, *p* < 0.01). Self-rated health exhibited a positive correlation with life satisfaction (*r* = 0.265, *p* < 0.01) and a negative correlation with depression (*r* = −0.399, *p* < 0.01). Furthermore, life satisfaction was negatively correlated with depression (*r* = −0.359, *p* < 0.01). Additional analyses suggest that self-rated health and life satisfaction act as significant mediators in the relationship between pain and depression. The mediation analysis revealed that the direct effect of pain, self-reported health, life satisfaction, and depression on the outcome was 62.13%, while the indirect effect accounted for 37.87%.

**Conclusion:**

The findings of this study contribute to a deeper understanding of the dynamics between pain and depression, providing essential insights for addressing depression in the middle-aged and older adult demographic.

## 1 Introduction

Depression is a widespread mood disorder marked by enduring feelings of sadness, diminished interest or pleasure, and a variety of physical and cognitive symptoms (1). According to the World Health Organization (WHO), around 280 million people globally are impacted by depression (2). The middle-aged and older adult demographic is identified as a high-risk group for this disorder. Research reveals that the global prevalence of depression among older adults stands at 28.4% (3). Secondly, depression can heighten the risk of suicide, particularly in the older adult population, where the suicide rate is notably elevated (4). Consequently, it is essential to raise awareness about depression in older adults and to enhance preventive and intervention strategies to protect their physical and mental health and to improve their quality of life.

Furthermore, according to the International Association for the Study of Pain, it is defined as an unpleasant sensory and emotional experience associated with actual or potential tissue damage, or is described in terms of such damage (5). Statistically, 60.02% of this population experiences chronic pain, with the most prevalent types being headaches, lower back pain, and knee pain (6). Older adults enduring pain are more likely to exhibit depressive symptoms compared to their counterparts who are pain-free (7). According to the biopsychosocial model, chronic pain can activate the body’s stress response, which may adversely affect sleep quality, subsequently leading to fatigue and diminished energy levels; these physiological alterations can heighten the risk of depressive states (8). Chronic pain imposes both physical and psychological stress, and this prolonged state of stress can trigger or intensify feelings of depression (9). Pain may limit individuals’ daily activities and social engagements, resulting in feelings of isolation and helplessness, thereby further amplifying the risk of depression (10, 11). Therefore, we propose the hypothesis that pain will have a significant impact on depression (Hypothesis 1: Pain → depression).

Additionally, self-rated health denotes an individual’s personal evaluation of their health condition. Research demonstrates that this subjective appraisal is frequently aligned with their actual physiological health status (12). Pain plays a significant role in shaping an individual’s self-rated health (13). Firstly, chronic or intense pain can adversely affect quality of life, disrupt daily functioning and sleep patterns, resulting in a diminished self-rated of health (14, 15). Secondly, ongoing pain may lead to increased utilization of healthcare services, thereby negatively impacting self-rated health from both psychological and physiological perspectives (16). Empirical studies indicate that individuals reporting poorer self-rated health are more prone to exhibit depressive symptoms (17). For example, they may withdraw from social engagements, experience feelings of helplessness and hopelessness, which can further intensify depressive states (18). Consequently, we infer that pain affects depression through self-rated health (Hypothesis 2: Pain → self-rated health → depression).

Finally, life satisfaction represents an individual’s comprehensive evaluation and perception of their quality of life, grounded in personal criteria, and is recognized as a vital metric for mental health and overall well-being (19). Research demonstrates a robust association between pain and diminished life satisfaction (20, 21). Investigations indicate that a reduction in life satisfaction may act as a precursor to the emergence of depressive moods and symptoms (22). When individuals perceive dissatisfaction in multiple life domains, they may encounter increased stress and negative emotional states, which could elevate the risk of depression (23). Sustained low life satisfaction may compromise an individual’s psychological resilience and coping mechanisms, rendering it more difficult to manage life’s adversities and stressors, thereby further amplifying the risk of depression (24). Consequently, we infer that pain affects depression through life satisfaction (Hypothesis 3: Pain → life satisfaction → depression).

Moreover, numerous investigations have demonstrated a significant correlation between individuals’ subjective assessments of their health and their overall life satisfaction (25, 26). Evidence suggests that those who perceive their health positively are more likely to report elevated levels of life satisfaction (27). Consequently, we infer that pain affects depression through self-rated health and life satisfaction (Hypothesis 4: Pain → self-rated health → life satisfaction → depression).

**Figure 1:**
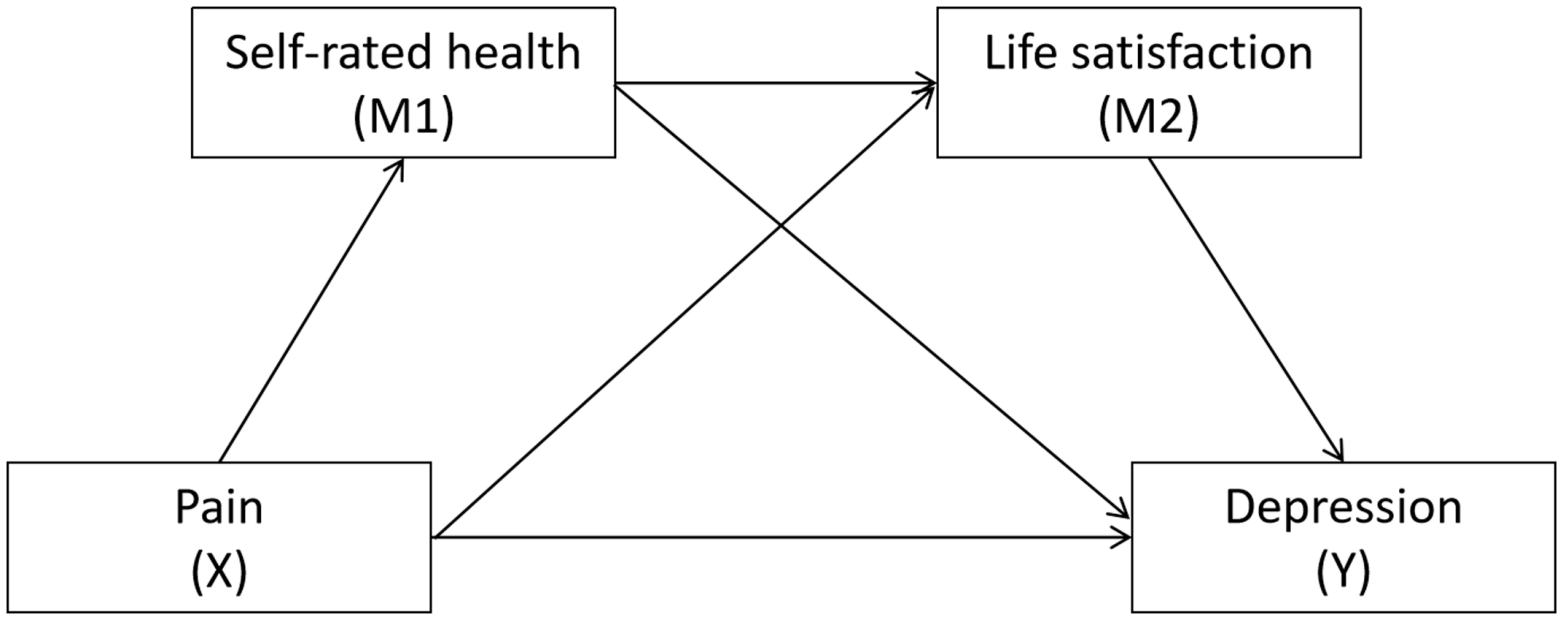
Hypothesized relationships between pain, self-rated health, life satisfaction and depression.

However, despite the valuable insights these investigations offer, a considerable knowledge gap persists in comprehending how these variables interact within more intricate systems. This gap hinders our capacity to formulate targeted interventions. Given the distinct challenges encountered by this demographic, it is essential to explore the interplay between pain, self-rated health, life satisfaction, and depression to enhance their physical and mental well-being. To bridge these research gaps and build upon prior findings, this study thoroughly investigates the interconnections among pain, self-rated health, and life satisfaction in middle-aged and older adults, as well as their collective impact on depression. In light of existing research, the specific hypotheses for this study are as follows (Figure 1).

## 2 Methods

### 2.1 Study subjects

The data for this study was derived from the 2020 China Health and Retirement Longitudinal Study (CHARLS) (28). Participants included individuals aged 45 and older, while those with incomplete primary research variables and individuals under 45 were excluded. A total of 19,395 middle-aged and older adults were selected as the target population, and after filtering out outliers and missing values by deleting the list, 16,054 individuals were ultimately included in the analysis. For categorical variables, we employed dummy variable coding. Specifically, sex was coded as (0 = women, 1 = men), age was categorized as (1 = 45–59 years, 2 = 60–74 years, 3 ≥ 75 years), marital status was coded as (0 = unmarried, 1 = married), residence was coded as (0 = urban, 1 = rural), educational attainment was categorized as (1 = not enrolled in school, 2 = elementary school, 3 = middle school, 4 = high school and above), and chronic diseases were categorized as (0 = none, 1 = prevalence). The 2020 CHARLS questionnaire included 15 chronic diseases such as hypertension, diabetes, and heart disease. Interviewers assessed whether respondents had been told by their doctors about these diseases. Each disease was scored as 1 point. Respondents were classified as having chronic diseases if their total score was ≥1.

### 2.2 Research variables

#### 2.2.1 Sociodemographic factors

This study has examined the impact of various sociodemographic factors on depression, including gender, age, marriage, residence, education, and chronic disease.

#### 2.2.2 Explanatory variables

The pain assessment questionnaire identifies 16 specific pain locations, including the head, shoulders, arms, wrists, etc. Participants were asked, “Which parts of your body experience pain? Please list all affected areas.” Each identified area was scored as 1 point, with a maximum total score of 16 points; a higher score indicates a greater number of painful areas.

#### 2.2.3 Mediating variables

The mediating variables in this study are self-rated health and life satisfaction. Self-rated health was assessed through the question, “How do you perceive your current health status?” Responses included “very good,” “good,” “fair,” “poor,” and “very poor,” assigned values of 5, 4, 3, 2, and 1, respectively, creating an ordinal variable where higher scores reflect better self-rated health among middle-aged and older adults. Life satisfaction was determined by the question, “How do you feel about your current life?” with responses of “extremely satisfied,” “very satisfied,” “somewhat satisfied,” “not very satisfied,” and “not satisfied at all,” assigned values of 5, 4, 3, 2, and 1, respectively, also forming an ordinal variable where higher scores indicate greater life satisfaction among this demographic.

#### 2.2.4 The dependent variable

Depression was assessed using the short version of the Center for Epidemiological Studies Depression Scale (CESD-10) from CHARLS. This scale employs a 4-point Likert scoring system, with responses categorized as “not at all or rarely” (0 points), “occasionally or seldom” (1 point), “sometimes or moderately” (2 points), and “often or always” (3 points). The maximum score is 30, with a CESD-10 score of ≥10 indicating the presence of depressive symptoms. Higher scores correlate with an increased risk of depression (29).

### 2.3 Statistical methods

Data cleaning, organization, and statistical analysis were conducted using SPSS 26.0 software. Continuous data are presented as mean ± standard deviation, while categorical data are expressed as counts or percentages. By applying linear regression models, we delved into the key factors influencing depression in middle-aged and older adult individuals. We used the Kolmogorov–Smirnov test to verify the normality of the data. Pain, self-rated health, life satisfaction, and depression were all consistent with a normal distribution. Pearson correlation analysis was utilized to explore the relationships between variables. The range of the correlation coefficient is [−1, 1], the greater the absolute value, the stronger the correlation between the two variables. The PROCESS 3.4 macro was employed to establish a mediation effect model, analyzing the mediating role of self-rated health and life satisfaction in the relationship between pain and depression in middle-aged and older adults. A Bootstrap method with 5,000 resamples was used to test the significance of the mediation effect. PROCESS 3.4 incorporated the advantages of linear regression and structural equation modeling, utilizing bootstrapping methods to estimate indirect effects, thus offering more precise results.

## 3 Result

### 3.1 The results of the linear regression analysis on the demographic characteristics of participants and their impact on depression

The most painful locations in this study were the waist and knees. Depression scores reached a mean of 8.63 ± 6.45. Through linear regression analysis, we identified that gender, age, marriage, residence, education and chronic disease significantly impact depression, as outlined in Table 1.

**Table 1:** Linear regression results of demographic characteristics and their impact on depression (*n* = 16,054).

Variables	*n* (%)	β	*t*	*p*
Gender		−0.139	−17.918	<0.001
Men	8,356 (52.05)			
Women	7,698 (47.95)			
Age (Y)		0.023	2.890	0.004
45 ~ 59	6,951 (43.30)			
60 ~ 74	7,389 (46.03)			
≥75	1714 (10.67)			
Marriage		−0.084	−10.888	<0.001
Married	13,828 (86.13)			
Other	2,226 (13.87)			
Residence		0.117	15.174	<0.001
Rural	9,600 (59.80)			
Urban	6,454 (40.20)			
Education		−0.157	−19.349	<0.001
Not enrolled in school	6,339 (39.49)			
Elementary school	3,677 (22.90)			
Middle school	3,840 (23.92)			
High school and above	2,198 (13.69)			
Chronic disease		0.155	20.762	<0.001
Yes	12,900 (80.35)			
No	3,154 (19.65)			

### 3.2 Pearson correlation analysis

The results of the Pearson correlation analysis indicate that in middle-aged and older adults, pain is negatively correlated with self-rated health and life satisfaction (*r* = −0.381, *p* < 0.01; *r* = −0.158, *p* < 0.01), and positive correlated with depression (*r* = 0.356, *p* < 0.01). Self-rated health showed a positive correlation with life satisfaction (*r* = 0.265, *p* < 0.01) and a negative correlation with depression (*r* = −0.399, *p* < 0.01). Additionally, life satisfaction was negatively correlated with depression (*r* = −0.359, *p* < 0.01). Refer to Table 2 for further details.

**Table 2:** Correlation between pain, self-rated health, life satisfaction, and depression among middle-aged and older adult individuals (r values).

Variable	1	2	3	4
1. Pain	1.000			
2. Self-rated health	−0.381**	1.000		
3. Life satisfaction	−0.158**	0.265**	1.000	
4. Depression	0.356**	−0.399**	−0.359**	1.000

** indicates *p* < 0.01.

### 3.3 Mediation effect analysis

**Figure 2:**
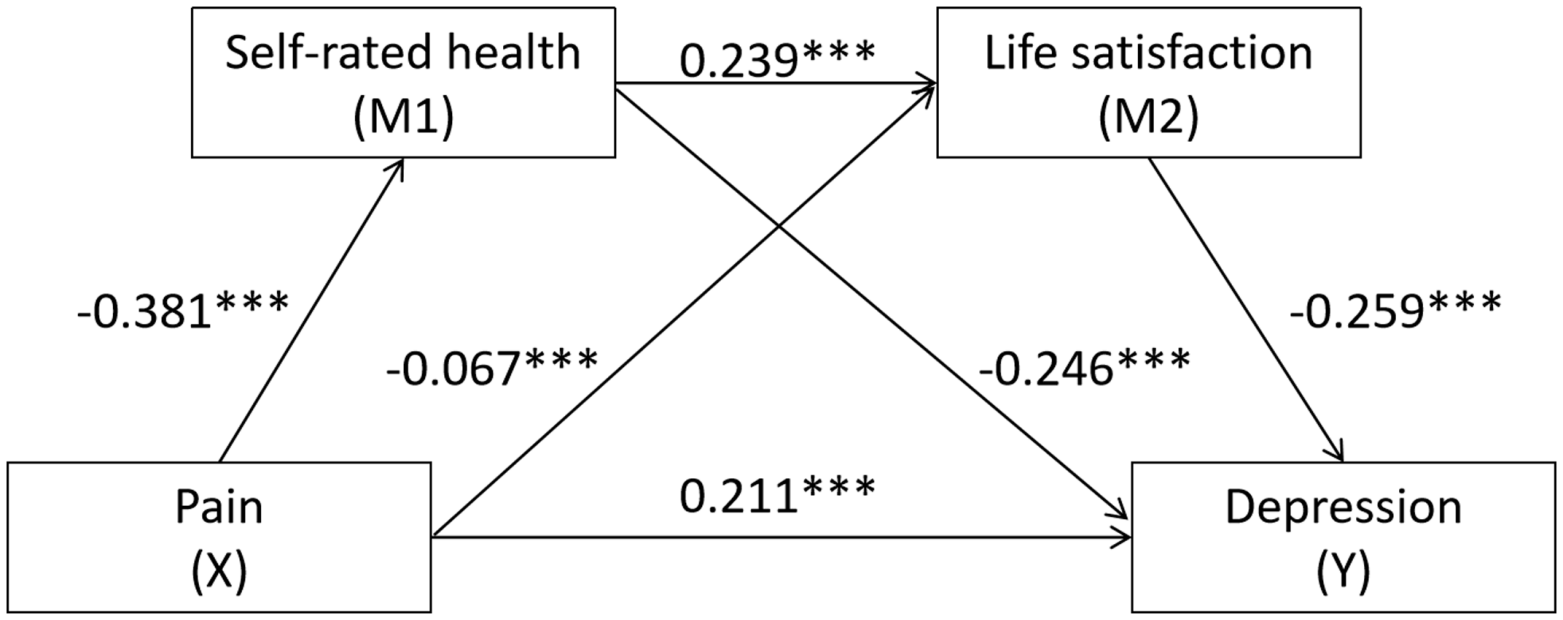
The chain mediating effect of pain and depression.*** indicates *p* < 0.001.

Mediation effect analysis was conducted with depression as the dependent variable, pain as the independent variable, and self-rated health and life satisfaction as mediating variables, utilizing a chain mediation approach. The results were illustrated in Figure 2. Pain, self-rated health, and life satisfaction predicted depression (*β* = 0.221, −0.246, −0.259, *p* < 0.001). For further details, please refer to Table 3. The bootstrap analysis, which was conducted to assess the mediating effects of pain on self-rated health, life satisfaction, and depression, reveals significant findings as presented in Table 4. The mediation analysis revealed that the direct effect of pain, self-rated health, life satisfaction, and depression on the outcome was 62.13%, while the indirect effect accounted for 37.87%.

**Table 3:** Direct effect test of pain, self-rated health, life satisfaction, and depression.

Path	*B*	β	SE	95%CI	*p*
X → M1	−0.117	−0.381	0.002	−0.121 ~ −0.112	<0.001
X → M2	−0.016	−0.067	0.002	−0.019 ~ −0.012	<0.001
X → Y	0.425	0.221	0.014	0.397 ~ 0.452	<0.001
M1 → M2	0.182	0.239	0.006	0.170 ~ 0.194	<0.001
M1 → Y	−1.548	−0.246	0.047	−1.640 ~ −1.456	<0.001
M2 → Y	−2.138	−0.259	0.058	−2.252 ~ −2.025	<0.001

*B = unstandardized regression coefficients, β = standardized regression coefficients. SE = standard error; Boot LLCI = bootstrapping lower limit confidence interval; Boot ULCI = bootstrapping upper limit confidence interval; X = pain; M1 = self-rated health; M2 = life satisfaction; Y = depression.*

**Table 4:** Bootstrap analysis of mediation effects of pain, self-rated health, life satisfaction, and depression.

Effect	Point of estimate	SE	95%CI	*p*	Relative mediation effect (%)
Direct effect	0.425	0.014	0.397 ~ 0.452	<0.001	62.13%
Indirect effect	0.259	0.008	0.243 ~ 0.276	<0.001	37.87%
Total effect	0.684	0.014	0.656 ~ 0.712	<0.001	100%
X → M1 → Y	0.180	0.007	0.168 ~ 0.193	<0.001	26.32%
X → M2 → Y	0.033	0.005	0.024 ~ 0.042	<0.001	4.82%
X → M1 → M2 → Y	0.045	0.002	0.041 ~ 0.050	<0.001	6.58%

## 4 Discussion

This research seeks to explore the interplay between pain and depression within the middle-aged and older adult demographic, emphasizing the mediating influences of self-rated health and life satisfaction. The study presents three primary conclusions. Firstly, a significant positive correlation exists between pain and depression. Secondly, self-rated health and life satisfaction serve as critical mediators in the connection between pain and depression. Lastly, pain exerts an indirect influence on the depression levels of older adults through the chain mediation of self-rated health and life satisfaction. The findings underscore a substantial positive association between pain experiences and depression levels among older adults. Increased pain severity was linked to heightened depression, thereby corroborating hypothesis H1 has been confirmed. This observation is consistent with prior studies that have validated the relationship between pain and depression (30). The biopsychosocial model posits that pain can induce emotional distress, anxiety, and feelings of helplessness, which contribute to depressive symptoms (8). Pain limits patients’ daily activities and social engagements, reduces quality of life, and intensifies psychological burdens. It may also lead to diminished work capacity, strained interpersonal relationships, and decreased social support, thereby elevating the risk of depression (8). The middle-aged and older adults may face challenges in pain management, which not only impacts their daily functioning but can also worsen depressive feelings. Consequently, it is imperative to prioritize pain management within this population, offering vital support and resources to improve their quality of life. Fostering a supportive social environment, coupled with pain education and self-management techniques, may assist older adults in the early detection and alleviation of their pain, thereby alleviating depressive symptoms and preventing further psychological complications (31).

The self-rated health was served as a mediating factor in the interplay between pain and depression within the middle-aged and older adult demographic. Pain may lead individuals to report health complications, which can subsequently intensify depressive symptoms, thereby supporting Hypothesis H2. Prior studies have highlighted pain as a significant determinant of an individual’s self-rated health (32). The stress-adaptation framework emphasizes pain as a stressor that can induce both physical and psychological distress, thereby influencing self-reported health status (33). Self-rated health pertains to an individual’s personal assessment of their health condition (34). When an individual perceives their health to be suboptimal, it can adversely affect their daily functioning, diminish social interactions, and potentially result in avoidance behaviors. This alteration in psychological state heightens the likelihood of experiencing depressive symptoms (35). Consequently, fostering a favorable self-assessment of health is essential for the prevention and mitigation of depressive symptoms, the enhancement of quality of life, and the promotion of comprehensive physical and mental well-being.

Life satisfaction has been identified as a mediating variable between pain and depression in the middle-aged and older adult demographic. As pain intensity escalates, life satisfaction tends to increase, subsequently aiding in the reduction of depressive symptoms. Consequently, the H3 hypothesis has been substantiated. This observation is consistent with research conducted in the United States, which similarly identified the mediating function of life satisfaction in the interplay between pain and depression among middle-aged and older adults (36). Studies reveal that individuals experiencing chronic pain generally report diminished levels of life satisfaction (37). The efficacy of pain management is intricately connected to patients’ life satisfaction (38). Effective pain alleviation can markedly improve quality of life and life satisfaction, while elevated life satisfaction plays a role in mitigating depressive symptoms (39). Given the mediating influence of life satisfaction in the nexus of pain and depression, it is crucial to implement targeted interventions aimed at enhancing life satisfaction. Approaches such as promoting social engagement, bolstering health management, and offering psychological support may enhance life satisfaction among middle-aged and older adult individuals, thereby improving overall mental health (39, 40).

The research demonstrates that pain has an indirect impact on the depression of middle-aged and older adults through a mediating chain effect that encompasses self-rated health and life satisfaction. As a result, Hypothesis H4 has been validated. The study reveals that prolonged or intense pain experiences adversely affect individuals’ self-evaluations of health, diminish positive life experiences, and lower overall life satisfaction, ultimately contributing to feelings of sadness and depression (13, 41). The older adult demographic frequently encounters more significant pain challenges due to the progressive decline in physical capabilities (42). With advancing age, they become increasingly vulnerable to chronic pain, which not only detracts from their quality of life but may also precipitate emotional issues such as depression (43, 44). The outcomes of this mediating model highlight the critical need for psychological health interventions that address not only the direct consequences of pain but also comprehensively consider the roles of mediating factors. Health education and practical interventions should focus on improving self-rated health and life satisfaction to effectively alleviate the influence of pain on depressive moods in middle-aged and older adults. By tackling these interrelated factors, organizations can enhance their support for the psychological health and overall well-being of the middle-aged and older adult. Moreover, motivated by the findings of this study, healthcare institutions should emphasize the early detection and intervention of pain in clinical settings, formulating targeted intervention strategies. Future research should persist in examining the efficacy of these targeted interventions in addressing these challenges within the healthcare framework. By prioritizing these initiatives, organizations can proactively bolster the psychological health of middle-aged and older adults and improve their overall well-being.

## 5 Limitations

This research has offered a novel insight into the interplay between pain and depression among middle-aged and older adults, examining the mediating influences of self-rated health and life satisfaction. Nonetheless, several limitations may impact the generalizability and interpretability of the findings. Firstly, this study utilizes the CHARLS dataset, which, despite its comprehensiveness, may present biases in regional and demographic selection, potentially failing to adequately represent the entirety of middle-aged and older adult populations. Future investigations could broaden the sample scope to encompass middle-aged and older adults from varied regions and cultural contexts to improve the generalizability and external validity of the results. Secondly, pain, self-rated health and life satisfaction are contingent upon subjective evaluations from participants, which may be swayed by individual cognitive and reporting biases. Subsequent studies should consider integrating more objective assessment techniques. The utilization of objective scales for life satisfaction assessment, along with the incorporation of objective health status and pain indicators (e.g., clinical assessments, biomarkers), may reduce reporting bias risks and offer a more comprehensive perspective on these relationships. Thirdly, the chain mediation effect involving self-rated health and life satisfaction as mediators may be influenced by other unexamined mediating factors. Meanwhile, the relationships between these variables are relatively small, indicating that despite the importance of pain and depression, their impact on self-reported health and life satisfaction is not strong. This suggests that other factors may have a more pronounced influence on these outcomes. Future research could investigate the potential presence of additional mediators and mixed factors, such as social support and economic status, which may affect the relationship between pain and depression. Fourthly, due to the cross-sectional design of the study, establishing the temporal sequence and causal relationships among pain, depression, and the mediating variables is not feasible. Future research could implement longitudinal study designs to clarify the temporal order and causal dynamics among these variables. Lastly, the research outcomes may be shaped by the specific cultural and social contexts of China, potentially limiting their applicability to middle-aged and older adult populations in other nations or cultural environments. Future studies could engage in comparative research across diverse cultural backgrounds to examine the cultural specificity of the relationship between pain and depression. In addition, Future research should include more detailed assessments of pain, such as pain intensity, duration, and type, to better understand how different aspects of pain influence depression. Through these proposed research avenues, we can enhance our comprehension of the association between pain and depression, thereby providing a scientific foundation for the formulation of effective prevention and intervention strategies.

## 6 Conclusion

The findings of the extensive analysis reveal that the interplay between pain and depression among the middle-aged and older adult demographic is notably affected by self-rated health and life satisfaction. By implementing strategies to enhance self-rated health and life satisfaction, we can significantly mitigate depressive symptoms in middle-aged and older adults. These research outcomes not only enrich our comprehension of pertinent theories but also offer actionable insights for healthcare practitioners, empowering them to develop more focused intervention programs that aim to enhance the mental well-being and overall quality of life for the middle-aged and older adult population.

## Data Availability

Publicly available datasets were analyzed in this study. This data can be found here: https://charls.pku.edu.cn/en/. The raw data supporting the conclusions of this article will be made available by the authors without undue reservation.
